# Unveiling heterogeneity in MSCs: exploring marker-based strategies for defining MSC subpopulations

**DOI:** 10.1186/s12967-024-05294-5

**Published:** 2024-05-15

**Authors:** Si Chen, Bowei Liang, Jianyong Xu

**Affiliations:** 1 Shenzhen University Medical School, Shenzhen University, Shenzhen, 518000 People’s Republic of China; 2 Shenzhen Key Laboratory of Reproductive Immunology for Peri-Implantation, Guangdong Engineering Technology Research Center of Reproductive Immunology for Peri-Implantation, Shenzhen Zhongshan Obstetrics & Gynecology Hospital (formerly Shenzhen Zhongshan Urology Hospital), Fuqiang Avenue 1001, Shenzhen, 518060 Guangdong People’s Republic of China; 3 Guangdong Engineering Technology Research Center of Reproductive Immunology for Peri-Implantation, Shenzhen, 518000 People’s Republic of China

**Keywords:** Mesenchymal stem/stromal cells, MSCs, Subpopulation, Cell markers, Identity

## Abstract

Mesenchymal stem/stromal cells (MSCs) represent a heterogeneous cell population distributed throughout various tissues, demonstrating remarkable adaptability to microenvironmental cues and holding immense promise for disease treatment. However, the inherent diversity within MSCs often leads to variability in therapeutic outcomes, posing challenges for clinical applications. To address this heterogeneity, purification of MSC subpopulations through marker-based isolation has emerged as a promising approach to ensure consistent therapeutic efficacy. In this review, we discussed the reported markers of MSCs, encompassing those developed through candidate marker strategies and high-throughput approaches, with the aim of explore viable strategies for addressing the heterogeneity of MSCs and illuminate prospective research directions in this field.

## Introduction

MSCs (mesenchymal stem/stromal cells) are heterogenous cell populations, residing in various tissues (such as bone marrow, umbilical cord, teeth, adipose, and so on). Differing from other types of adult stem cells or terminal differentiated cells, the main function of MSCs is sensing and responding to micro-environmental disturbances. Due to their innate characteristics and functions, they have multiple ways to respond to micro-environmental changes, such as extracellular matrix modification, recruiting other cells (the immune cells, for example), secreting small factors with various functions (immune modulation and regeneration, for example) [1]. It is well-known that many diseases are resulting from micro-environment dysfunctions. Therefore, the MSCs have been intensively and extensively applied in treating different kinds of diseases. Both pre-clinical and clinical investigations have shown that the MSCs hold great promise in developing one new therapeutic approach for treating many kinds of diseases [1–5].

Soon after the first demonstration of MSCs, its therapeutic applications have been investigated for decades. Unfortunately, in contrast to the rapid growth of clinical trials, few of them eventually have been developed as applicable therapeutic products. In addition to other factors inducing the therapeutic inconsistency of MSCs, cell heterogeneity is one tough challenge in their way to achieve the expected clinical outcomes [1, 2, 4, 6, 7].

**Fig. 1:**
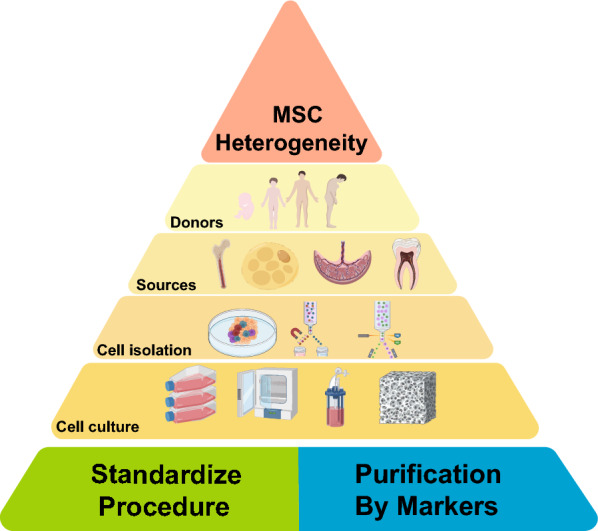
Illustration of factors inducing MSC heterogeneity and potential solutions. The MSC heterogeneity results from various factors, including donor conditions (age, gender, health conditions), tissue origin (bone, fat, placenta/umbilical cord, teeth), and the methods employed for isolating (plastic adherence, MACS, FACS) and expanding MSCs (2D, 3D bioreactor, 3D matrix). To address MSC heterogeneity and enhance their therapeutic stability, three primary strategies are currently employed. These strategies encompass standardizing the MSC production procedures and purifying MSC subpopulations by markers. *MSC* mesenchymal stem/stromal cell, *MACS* magnetic-activated cell sorting, *FACS* fluorescence-activated cell sorting, *2D* 2 dimensional, *3D* 3 dimensional

The heterogeneity of MSCs is reflected in different levels, such as the molecular levels (transcriptomics, proteomics, secretomics, and epigenomics), and the function levels (tri-lineage differentiation potentials, immunomodulatory capabilities, and regenerative activities) [2, 7, 8]. The heterogeneity of MSCs could be induced by various factors including the donor conditions (age, gender, health condition, genetic background, and so on), tissue origin, and the strategies to isolate and expand the MSCs (digestion enzyme, matrix protein, cell culture medium, passage number, and so on) [2, 6, 7, 9–12] (Fig. 1). The causes of MSC heterogeneity have been extensively described in the preceding reviews, and we will not delve into them further. Pluripotent stem cell derived MSC, which can avoid the heterogeneity induced by the aforementioned factors, is also an important category of MSCs [13, 14]. However, we will focus exclusively on MSCs derived from somatic cells under natural conditions in this review.

**Fig. 2:**
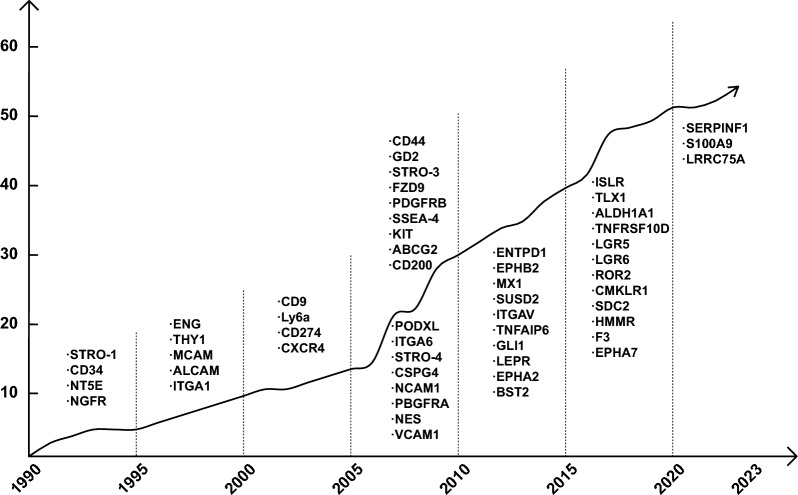
Timeline of MSC marker identification

Among different strategies to reduce the heterogeneity and improve the therapeutic consistency of MSCs, purifying the homogenous MSC subpopulations is suggested to yield more consistent clinical outcomes [6]. MSC subpopulations refer to distinct groups or subsets within the broader MSC population that are identified based on specific characteristics or markers. These characteristics can include surface protein expressions, functional properties, gene expression profiles, or responsiveness to different environmental cues. According to the minimal criteria for defining MSCs, stated by the International Society for Cellular Therapy in 2006 [15], 55 MSC markers have been identified so far from different tissues and species (Fig. 2, Table 1).

**Table 1:** Basic information for MSC markers

Name	Gene ID	Aliases	Full name	Year published	Positive (%)	Tissue	Species	References
STRO-1			Antibody recogniziing unknown target	1991	5–66.5	Bone marrow	Human	2070060
				2004	6	Bone marrow	Human	14715641
				2009	6	Bone marrow	Human	19143868
				2010	13.3	Bone marrow	Human	20162565
				2011	n.d	Adipose tissue	Rat	21903091
				2011	n.d	Bone marrow	Rat	21208041
				2011	n.d	Heart	Rat	21208041
				2011	n.d	Leg muscle	Rat	21208041
				2011	n.d	Aorta	Rat	21208041
				2011	n.d	Rectum	Rat	21208041
				2011	n.d	Adipose tissue	Human	21208041
				2011	n.d	Prostate tissue	Human	21208041
				2017	11	Tooth germ	Human	28686984
				2019	1.0–9.6	Gingiva	Human	30260000
				2021	10	Dental follicle	Human	33278647
				2021	15	Periodontal ligament	Human	33278647
				2021	20	Dental pulp	Human	33278647
CD34	947		CD34 molecule	1991	n.d	Bone marrow	Human	1720038
				2007	1.8	Bone marrow	Human	17786605
				2016	n.d	Placental amnion membrane	Human	27405780
NT5E	4907	NT; eN; NT5; NTE; eNT; CD73; E5NT; CALJA;	5'-nucleotidase ecto	1992	n.d	Bone marrow	Human	1316137
				2006	n.d	Chondrocyte	Murine	16443378
				2006	n.d	Osteoblast precursors	Human	16418778
				2008	n.d	Bone marrow	Human	18086871
				2017	44.8–69.1	Bone marrow	Murine	28684854
				2018	n.d	Bone marrow	Murine	29451855
				2019	n.d	Bone marrow	Murine	31279774
				2021	n.d	Adipose tissue	Human	33407847
NGFR	4804	CD271; p75NTR; TNFRSF16; p75(NTR); Gp80-LNGFR;	Nerve growth factor receptor	1993	n.d	Bone marrow	Human	7681701
				2006	n.d	Bone marrow	Human	16977637
				2007	n.d	Bone marrow	Human	17395729
				2010	0.2–2.5	Bone marrow	Human	20179086
				2012	n.d	Bone marrow	Human	22268519
				2012	n.d	Bone marrow	Human	22048731
				2012	5.5	Adherent dermal	Human	22048731
				2012	31.4	Embryonic face	Rat	22982680
				2015	10.6	Dental pulp	Human	26674422
				2018	0.1–1.1	Bone marrow	Human	29482445
				2018	3.8–13	Bone marrow	Human	29915318
				2018	1.5–5.9	Adipose tissue	Human	29915318
				2018	0–0.5	Amniotic fluid	Human	29915318
				2018	0–0.5	Cord blood	Human	29915318
				2019	n.d	Adipose tissue	Human	30816233
				2021	19.1–22.1	Adipose tissue	Human	33653407
ENG	2022	END; HHT1; ORW1;	Endoglin	1996	n.d	Bone marrow	Human	8652367
				2000	98	Bone marrow	Human	10942523
				2005	33.7–46.5	Bone marrow from young	Human	16507351
				2005	36.7–43.9	Bone marrow from old	Human	16507351
				2006	1.9–2.8	Bone marrow	Human	16601078
				2010	1.2–82.0	Adipose tissue	Human	20153525
				2011	n.d	Synovium	Rat	21205995
ALCAM	214	MEMD; CD166;	Activated leukocyte cell adhesion	1997	n.d	Bone marrow	Human	9276087
				1998	n.d	Bone marrow	Rat	9556065
				1998	n.d	Bone marrow	Rabbit	9556065
				1998	n.d	Bone marrow	Canine	9556065
				1998	n.d	Bone marrow	Human	9556065
				2002	5.7–91.4	Bone marrow	Murine	12070283
				2011	11.5–72.2	Cartilage	Human	21787134
				2011	n.d	Bone marrow	Horse	21782255
				2011	n.d	Adipose tissue	Horse	21782255
				2015	n.d	Amniotic membrane	Porcine	26540004
				2020	n.d	Bone marrow	Human	34493362
				2020	n.d	Umbilical cord	Human	34493362
MCAM	4162	CD146; MUC18; HEMCAM; METCAM; MelCAM;	Melanoma cell adhesion molecule	1998	n.d	Bone marrow	Human	9529137
				2003	n.d	Bone marrow	Human	12674330
				2003	n.d	Dental pulp	Human	12674330
				2007	n.d	Bone marrow	Human	17332507
				2007	n.d	Dental pulp	Human	17332507
				2007	1.3–1.7	Endometrial tissue	Human	17872908
				2011	n.d	Bone marrow	Human	21415267
				2016	n.d	Bone marrow	Human	26753846
				2016	12–25	Umbilical cord	Human	26841872
				2016	14.7–99.4	Bone marrow	Human	26941359
				2016	39.5	Periapical cyst	Human	27406247
				2019	60–90	Bone marrow	Human	31002939
				2020	34.6–65.6	Bone marrow	Human	32379908
				2021	11	Gingival tissue	Human	33777147
				2021	n.d	Dental pulp	Human	34461987
				2022	70–80	Umbilical cord	Human	35729643
THY1	7070	CD90; CDw90;	Thy-1 cell surface antigen	1999	n.d	Bone marrow	Human	10102814
				2009	n.d	Bone marrow	Murine	19841085
				2009	95–100	Bone marrow	Human	18985728
				2009	32–75	Bone marrow	Human	18985728
				2009	38–96	Amnion	Human	18985728
				2009	63–91	Chorion	Human	18985728
				2014	n.d	Incisor tooth	Murine	25079316
				2016	98	Dental pulp	Human	27465541
				2016	98	Adipose tissue	Human	27465541
				2016	98	Amniotic fluid	Human	27465541
				2018	30	Incisor pulp	Murine	29371677
				2018	8.8–10.2	Arterie	Human	30008326
				2019	n.d	Cardiac	Murine	31353772
ITGA1	3672	VLA1; CD49a;	Integrin subunit alpha 1	2000	22–89	Bone marrow	Human	10911362
				2003	2.2–4.0	Bone marrow	Human	12877680
				2003	55	Bone marrow	Human	12883998
				2005	2.5–26.8	Bone marrow	Human	15676216
				2007	16.8	Bone marrow	Human	17694277
				2007	3.2–4.0	Bone marrow	Human	17109120
				2007	4–5	Bone marrow	Murine	17109120
				2007	1.0–1.2	Bone marrow	Rat	17109120
CD9	928	MIC3; MRP-1; BTCC-1; DRAP-27; TSPAN29; TSPAN-29;	CD9 molecule	2001	20–36	Adipose tissue	Human	11573204
				2007	18.4–32.6	Adipose tissue	Human	17668233
Ly6a	110,454	TAP; Sca1; Sca-1; Ly-6A.2; Ly-6A/E; Ly-6E.1;	Lymphocyte antigen 6 family member A	2003	n.d	Bone marrow	Murine	12732718
				2003	n.d	Bone marrow	Murine	14616976
				2008	75.0–90.6	Ear	Murine	18599810
				2017	n.d	Bone marrow	Murine	27734598
				2021	20.3	Lung	Murine	34341173
				2022	n.d	Lung	Murine	35445270
CXCR4	7852	FB22; HM89; LAP3; LCR1; NPYR; WHIM; CD184; LAP-3; LESTR; NPY3R; NPYRL; WHIMS; HSY3RR; NPYY3R; WHIMS1; D2S201E;	C-X-C motif chemokine receptor	2004	Cell surface: 0–1; Intracellular: 83–98	Bone marrow	Human	15251986
				2006	30–56	Bone marrow	Human	16253981
				2006	11.5–21.6	Cord blood	Human	16410389
				2006	14.7–21.5	Bone marrow	Human	16410389
				2007	87.4–97.8	Bone marrow	Human	17606439
				2008	n.d	Bone marrow	Human	18334485
				2008	Cell surface: 0.5–4.1; Intracellular: 51–75	Bone marrow	Human	18728032
				2012	Cell surface: 20.9–25.1; Intracellular: 71.8–83.2	Fetal blood	Human	23197643
				2014	90–100	Bone marrow	Rat	24626964
				2014	8.4–11.0	Umbilical cord	Rat	25098450
				2017	n.d	Bone marrow	Murine	28352314
				2020	15.0–34.4	Bone marrow	Murine	32418119
CD274	29,126	B7-H; B7H1; PDL1; PD-L1; hPD-L1; PDCD1L1; PDCD1LG1;	CD274 molecule	2005	n.d	Bone marrow	Murine	15827960
				2008	n.d	Bone marrow	Murine	18607390
				2020	n.d	Bone marrow	Murine	32509271
				2020	n.d	Gingiva tissue	Human	32707035
CD44	960	IN; LHR; MC56; MDU2; MDU3; MIC4; Pgp1; CDW44; CSPG8; H-CAM; HCELL; ECM-III; HUTCH-1; HUTCH-I; ECMR-III; Hermes-1;	CD44 molecule	2006	n.d	Bone marrow	Murine	16306150
				2006	25.5–39.1	Ap8c3	Rat	16306150
				2007	n.d	Bone marrow	Murine	17507906
				2012	38–52	Bone marrow	Murine	22654106
				2013	n.d	Bone marrow	Human	23847000
				2018	n.d	Neural crest	Human	29571051
GD2				2007	95	Bone marrow	Human	17264296
STRO-3			Antibody recogniziing TNSALP(tissue nonspecific alkaline phosphatase, a cell-surface glycoprotein)	2007	n.d	Bone marrow	Human	8158854
				2009	n.d	Bone marrow	Sheep	19231391
				2010	n.d	Bone marrow	Sheep	20850099
				2011	n.d	Bone marrow	Human	21155976
				2012	n.d	Bone marrow	Sheep	22404141
				2013	n.d	Bone marrow	Sheep	23658436
				2017	n.d	Bone marrow	Sheep	28173831
				2021	n.d	Bone marrow	Human	33045417
FZD9	8326	FZD3; CD349;	Frizzled class receptor 9	2007	n.d	Placenta	Human	17288545
				2007	n.d	Bone marrow	Human	17288545
				2008	n.d	Placenta	Human	17924962
				2011	n.d	Placenta	Human	20658518
PDGFRB	5159	IMF1; KOGS; IBGC4; JTK12; PDGFR; PENTT; CD140B; PDGFR1; PDGFR-1;	Platelet derived growth factor receptor beta	2007	69.0–74.2	Endometrial tissue	Human	17872908
SSEA-4			Stage-specific embryonic antigen-4	2007	71	Bone marrow	Murine	17062733
				2010	33.3	Periodontal ligament	Human	19945209
				2012	22.7	Periodontal ligament	Human	22895512
				2012	45.5	Dental pulp	Human	22266579
				2013	11–99.6	Bone marrow	Human	23330736
				2014	n.d	Adipose tissue	Human	25123923
				2017	70–86	Bone marrow	Human	29078802
KIT	3815	PBT; SCFR; C-Kit; CD117; MASTC;	KIT proto-oncogene, receptor tyrosine kinase	2007	n.d	Adipose tissue	Human	17348807
				2014	0.5	Adipose tissue	Human	24713343
				2014	n.d	Adipose tissue	Murine	24713343
ABCG2	9429	MRX; MXR; ABCP; BCRP; BMDP; MXR1; ABC15; BCRP1; CD338; GOUT1; MXR-1; CDw338; CDw388; UAQTL1; EST157481;	ATP binding cassette subfamily G member 2	2011	n.d	Lung	Murine	21312316
CD200	4345	MRC; MOX1; MOX2; OX-2;	CD200 molecule	2008	n.d	Bone marrow	Human	18086871
				2012	7.5–69.6	Bone marrow	Human	22363701
				2012	0.4–0.5	Umbilical cord blood	Human	22363701
				2012	90	Heart	Human	22575528
				2012	25	Bone marrow	Human	22575528
				2012	0–10	Adipose tissue	Human	22575528
				2014	70.5	Full-term placenta (fetal origin)	Human	24721710
				2014	1.8	Full-term placenta (maternal origin)	Human	24721710
				2016	23–63.4	Bone marrow	Human	26773707
				2017	80	Bone marrow	Murine	28295880
PODXL	5420	PC; PDX; PCLP; Gp200; gp135; PCLP-1; PODXL1;	Podocalyxin like	2009	n.d	Bone marrow	Human	18818395
ITGA6	3655	JEB6; CD49f; VLA-6; ITGA6A; ITGA6B;	Integrin subunit alpha 6	2009	n.d	Bone marrow	Human	18818395
				2012	n.d	Umbilical cord blood	Human	22311737
				2013	n.d	Bone marrow	Human	23132820
				2013	n.d	Umbilical cord blood	Human	23132820
				2015	45.7–78.5	Fetal bone marrow	Human	26013602
				2015	11	Adult bone marrow	Human	26013602
				2020	n.d	Aorsal skin	Murine	31494092
				2021	5.3–17.7	Adipose tissue	Rat	33704842
STRO-4			Antibody recogniziing Hsp90β	2009	99.9	Bone marrow	Human	19327008
				2009	92.3	Adipose tissue	Human	19327008
				2009	95	Dental pulp	Human	19327008
				2009	86.2	Periodontal ligament	Human	19327008
				2009	99.9	Bone marrow	Sheep	19327008
				2009	91.9	Adipose tissue	Sheep	19327008
				2009	99.9	Dental pulp	Sheep	19327008
				2009	99.4	Periodontal ligament	Sheep	19327008
CSPG4	1464	NG2; MCSP; MCSPG; MSK16; CSPG4A; HMW-MAA; MEL-CSPG;	Chondroitin sulfate proteoglycan 4	2009	13.3–89.8	Bone marrow	Human	19462316
				2013	n.d	Bone marrow	Murine	24107994
				2013	95–100	Bone marrow	Human	23611563
NCAM1	4684	CD56; NCAM; MSK39;	Neural cell adhesion molecule 1	2009	n.d	Bone marrow	Human	19066333
				2016	22.8–95.9	Bone marrow	Human	27528376
				2019	1–35	Bone marrow	Human	30676001
PDGFRA	5156	CD140A; PDGFR2; PDGFR-2;	Platelet derived growth factor receptor alpha	2009	n.d	Bone marrow	Murine	19841085
				2012	5–10	Bone marrow	Murine	23154782
				2013	79–85	Bone marrow	Human	23776077
				2014	10–73	Muscle	Human	24743741
				2014	n.d	Bone marrow	Human	25454633
				2018	n.d	White adipose tissue	Murine	29378823
				2018	n.d	Bone marrow	Murine	29378823
				2018	6.8	Bone marrow	Murine	29529192
NES	10,763	Nbla00170	Nestin	2010	n.d	Bone marrow	Murine	20703299
				2013	n.d	Bone marrow	Human	23776077
				2015	n.d	Kidney	Murine	25736496
				2019	1.8–2.3	Bone marrow	Murine	31029167
				2020	15.3–18.5	Heart	Murine	31991111
				2020	3.7–4.6	Bone marrow	Murine	31991111
VCAM1	7412	CD106; INCAM-100;	Vascular cell adhesion molecule 1	2010	n.d	Bone marrow	Murine	20130212
				2013	65	Placental chorionic villi	Human	23555021
				2013	32	Bone marrow	Human	23555021
				2013	7.4	Umbilical cord	Human	23555021
				2013	0.7	Adipose tissue	Human	23555021
				2013	n.d	Bone marrow	Human	24052950
				2016	57.5–68.3	Placenta chorionic villi	Human	27044487
				2020	n.d	Umbilical cord	Human	32597552
				2022	n.d	Umbilical cord	Human	35768999
ENTPD1	953	CD39; SPG64; ATPDase; NTPDase-1;	Ectonucleoside triphosphate diphosphohydrolase 1	2011	n.d	Bone marrow	Murine	21176405
				2013	n.d	Synovial membrane	Human	23804221
				2014	84.3	Bone marrow	Human	24043462
				2017	n.d	Gingiva tissue	Human	28210258
				2019	n.d	Gingiva tissue	Human	31076346
				2020	n.d	Gingiva tissue	Human	32565049
EPHB2	2048	DRT; EK5; ERK; CAPB; Hek5; PCBC; EPHT3; Tyro5; BDPLT22;	EPH receptor B2	2011	n.d	Bone marrow	Human	21056708
				2013	n.d	Bone marrow	Human	23413357
				2013	n.d	Bone marrow	Human	23711177
MX1	4599	MX; MxA; IFI78; IFI-78 K; lncMX1-215;	MX dynamin like GTPase 1	2012	n.d	Compact bone	Murine	22385654
SUSD2	56,241	W5C5; BK65A6.2;	Sushi domain containing 2	2012	3.6–4.8	Endometrial tissue	Human	22469435
				2013	n.d	Bone marrow	Human	23406305
				2021	5.1	Placenta	Human	33961124
ITGAV	3685	CD51; MSK8; VNRA; VTNR;	Integrin subunit alpha V	2013	16	Bone marrow	Human	23776077
				2013	76–82	Bone marrow	Murine	23776077
				2015	2.4–24	Periodontal ligament	Human	26674423
				2019	1.4	Bone marrow	Murine	31747966
				2021	13.4	Heart	Murine	33968928
TNFAIP6	7130	TSG6; TSG-6;	TNF alpha induced protein 6	2014	n.d	Bone marrow	Human	25385603
				2022	13.7–92.3	Bone marrow	Murine	36153571
				2022	n.d	Placenta	Murine	36153571
				2022	n.d	Adipose tissue	Murine	36153571
GLI1	2735	GLI; PPD1; PAPA8;	GLI family zinc finger 1	2014	79.9–80.1	Incisor pulp	Murine	24506883
				2015	n.d	Craniofacial bone	Murine	25799059
				2015	32	Bone marrow	Murine	25465115
				2017	n.d	Bone marrow	Human	28457748
				2017	n.d	Bone marrow	Murine	28457748
				2020	n.d	Periodontal ligament	Murine	32652075
				2020	n.d	Pulp tissue	Murine	32783935
				2020	n.d	Lung	Human	33046884
				2022	n.d	Bone marrow	Murine	36092701
LEPR	3953	OBR; OB-R; CD295; LEP-R; LEPRD;	Leptin receptor	2014	0.3	Bone marrow	Murine	24953181
				2016	0.2–0.4	Bone marrow	Murine	27053299
				2018	n.d	Bone marrow	Murine	33221380
EPHA2	1969	ECK; CTPA; ARCC2; CTPP1; CTRCT6;	EPH receptor A2	2015	n.d	Bone marrow	Human	25684225
				2015	n.d	Adipose tissue	Human	25684225
				2018	n.d	Bone marrow	Human	29941036
				2015	45.0–80.7	Placenta	Human	26700997
				2015	n.d	Umbilical cord	Human	26700997
				2018	n.d	Umbilical cord	Human	30342659
				2020	n.d	Wharton's Jelly	Human	32899389
BST2	684	CD317; HM1.24; TETHERIN;	Bone marrow stromal cell antigen 2	2015	1–3	Bone marrow	Human	26070611
				2022	n.d	Bone marrow	Human	35734183
ISLR	3671	Meflin; HsT17563;	Immunoglobulin superfamily containing leucine rich repeat	2016	n.d	Bone marrow	Murine	26924503
TLX1	3195	TCL3; HOX11;	T cell leukemia homeobox 1	2016	n.d	Bone marrow	Murine	27939685
				2019	n.d	Bone marrow	Murine	31320650
ALDH1A1	216	ALDC; ALDH1; HEL-9; HEL12; PUMB1; ALDH11; RALDH1; ALDH-E1; HEL-S-53e;	Aldehyde dehydrogenase 1 family member A1	2017	n.d	Adipose tissue	Human	28233376
				2023	n.d	Adipose tissue	Human	37261440
TNFRSF10D	8793	DCR2; CD264; TRUNDD; TRAILR4; TRAIL-R4;	TNF receptor superfamily member 10d	2017	20–35	Bone marrow	Human	28962588
LGR6	59,352	GPCR; VTS20631;	Leucine rich repeat containing G protein-coupled receptor 6	2017	n.d	Lung	Murine	28886383
LGR5	8549	FEX; HG38; GPR49; GPR67; GRP49;	Leucine rich repeat containing G protein-coupled receptor 5	2017	n.d	Lung	Murine	28886383
ROR2	4920	BDB; BDB1; NTRKR2;	Receptor tyrosine kinase like orphan receptor 2	2017	6.7–40.9	Bone marrow	Human	28833807
CMKLR1	1240	DEZ; ERV1; RVER1; ChemR23; CHEMERINR;	Chemerin chemokine-like receptor 1	2017	n.d	Bone marrow	Murine	27733019
				2022	n.d	Bone marrow	Human	35365767
				2022	n.d	Bone marrow	Human	35723360
SDC2	6383	HSPG; CD362; HSPG1; SYND2;	Syndecan 2	2018	0.1–0.2	Bone marrow	Human	29979191
				2020	n.d	Umbilical cord	Human	32169108
				2020	n.d	Umbilical cord	Human	33158246
HMMR	3161	CD168; IHABP; RHAMM;	Hyaluronan mediated motility receptor	2019	1.3–1.8	Umbilical cord	Human	31068579
F3	2152	TF; TFA; CD142;	Coagulation factor III; tissue factor	2020	20	Umbilical cord	Human	32252818
				2023	9.5	Wharton's Jelly	Human	36504438
EPHA7	2045	EHK3; EK11; EHK-3; HEK11;	EPH receptor A7	2020	5–20	Peripheral tissues	Murine	31471947
SERPINF1	5176	OI6; OI12; PEDF; EPC-1; PIG35;	Serpin family F member 1	2022	n.d	Lung	Murine	35445270
S100A9	6280	MIF; NIF; P14; CAGB; CFAG; CGLB; L1AG; LIAG; MRP14; 60B8AG; MAC387; S100-A9;	S100 calcium binding protein A9provided	2023	9.5	Wharton's Jelly	Human	36504438
LRRC75A	388,341	FAM211A; C17orf76;	Leucine rich repeat containing 75A	2023	n.d	Bone marrow	Human	37263619

''n.d.' indicates 'not determined'

**Fig. 3:**
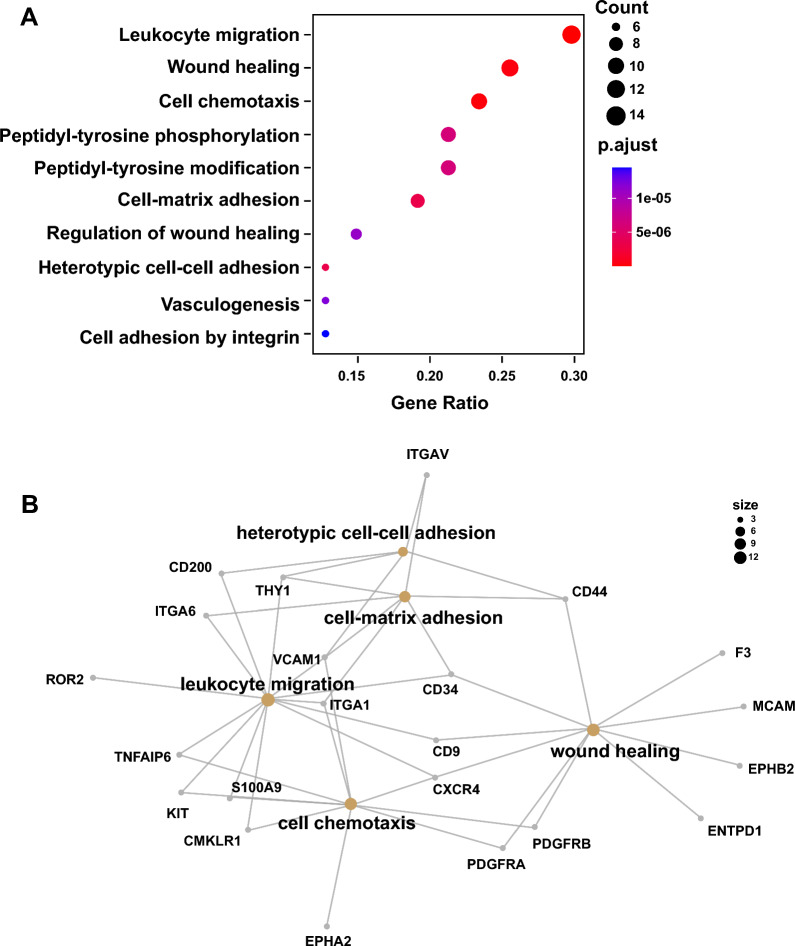
GO analysis of MSC markers. The bioinformatic analysis of GO enrichment of MSC markers was performed with Dotplotting (**A**), Cnetplotting (**B**). GO, gene ontology. *MSC* mesenchymal stem/stromal cell

**Fig. 4:**
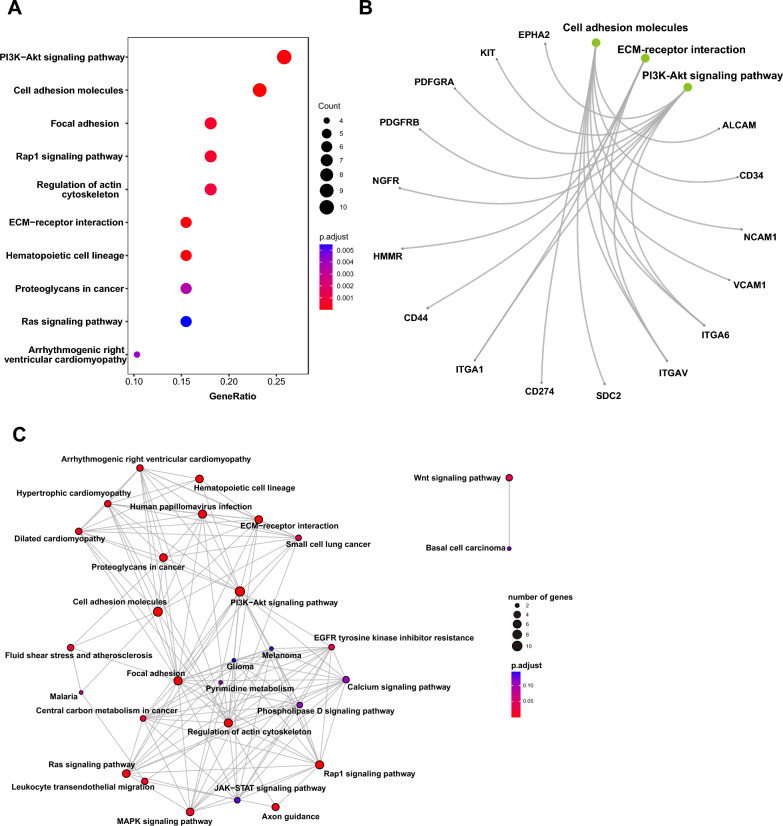
KEGG analysis of MSC markers. The bioinformatic analysis of KEGG enrichment of MSC markers was performed with Dotplotting (**A**), Cnetplotting (**B**), and Emapplotting (**C**). Bioinformatic analysis was conducted with package ‘enrichplot’ in R. KEGG, kyoto encyclopedia of genes and genomes. *MSC* mesenchymal stem/stromal cell

Function enrichment by GO (Gene Ontology) analysis indicates that these MSC markers mainly regulate the process of leukocyte migration, wound healing, cell chemotaxis, and so on (Fig. 3A). Although some markers are involved in multiple functions, some of them are also cross-interacted in a network way (Fig. 3B). KEGG (Kyoto Encyclopedia of Genes and Genomes) also indicates that these MSC markers are mainly involved in the signal pathways in PI3K-AKT, adhesion, and so on (Fig. 4A). Similar to the GO analysis, some markers regulated multiple pathways (Fig. 4B) and they are cross-interacted (Fig. 4C). Most of these MSC markers are localized on the cell membrane, which is suitable for cell purification with FACS (fluorescence-activated cell sorting) and MACS (magnetic-activated cell sorting), while some of them are also intracellularly or extracellularly localized (Table 2). Normally, MSCs enriched with specific makers have functional advantages (Table 2). However, in some cases, these enriched MSCs also have some disadvantages (Table 2).

**Table 2:** Pros and cons of MSC markers

Markers	Cellular localization	Generation	Pros	Cons	Refs
TNFAIP6	Extracellular (Secreted)	1st	Efficacy predictor of MSCs in treating inflammation	Low purfiying efficiency	25385603 36153571
			Higher immune suppression activities improved therapeutic effects		
			Improved therapeutic effects		
ALDH1A1	Intracellular (Cytosol)	1st	More primitive cell proliferation and tri-differentiation capabilites		37261440
STRO-1	Antibody recognizing unknown target	1st	Multilineage differentiation capabilities	Heterogenous	19143868 14715641 33278647 21903091 28686984 30260000
			Higher level of colony-forming activities	Expression level declines during passaging	
			Higher level of proliferative rate	Not specific,also expressed in endothelium	
			Higher level of immune suppression		
			Better homing activities		
			Expression level increases significantly during osteogenic differentiation		
ALCAM	Membrane	1st	Purify human MSCs from fibroblasts	Not specific, expressed in other types of cells	7535342 7760007 21787134 23280653 34493362
			More prone to differentiate into chondrocytes		
NT5E	Membrane	1st	Identify the MSCs in different organs in vivo	Expression level decreases during the differentiation process	16443378 16418778 18086871 29451855 31279774 28684854 33407847 34055772
			Higher level of colony-forming capabilities		
			Higher immune suppression activities		
			Much higher tri-differentiation abilities		
			Higher level of regeneration cytokines		
			Improved therapeutic effects		
			Much more smaller with spindle and rod-like shapes		
ITGA1	Membrane	1st	Stronger colony-forming activity	Expression levels upregulated during osteogenic differentiation	10911362 12877680 17694277 17109120 12883998
			Stronger differentiation capabilities	Not specific, also expressed in myofibroblasts	
			Higher expression levels of THY-1 and ENG		
CXCR4	Membrane	1st	Contributes to MSCs homing	Expression level is low on the cell surface	15251986 17606439 12357350 15837815 18728032 23197643 11283404 15153618 24626964 25098450 16410389 32418119
			Enhanced homing activities	Expression level decreases during passaging	
				Expression level decreases during aging	
CD9	Membrane	1st	Higher NOS (nitric oxide synthase) expression	Expression level declines during in vitro expansion	17668233 30356731
			Higher proliferation rate	Not specific,also expressed in lymphocytes	
			Higher colony formation ratio		
			Stronger cell adhesion capability		
			Better engraftment		
			Improved therapeutic effect		
CD44	Membrane	1st	Contributes to the MSC recruitment	Gradually up-regulated during cell expansion	16306150 17507906 29571051 22654106 23847000
			Contributes to migration and adhesion of MSCs	Widely expressed in multiple types of cells	
			Enhanced colony-forming capacity	Very low level in bone marrow MSCs	
			Enhanced in vitro differentiation abilities		
GD2	Membrane	1st	Much higher proliferation		17264296
			Higher colony formation capabilities		
			Better differentiation abilities		
MCAM	Membrane	1st	Enhanced colony formation capabilities	Expression declines during in vitro expansion	17956733 17872908 26753846 31002939 33777147 26841872 34461987 35729643 32379908 24188055 26941359 31070478
			Much higher colony-forming	Expressed in many cell type	
			Much stronger chemotactic attraction		
			Enhanced immune suppression activities		
			Enhanced immune suppression and secretory capacities		
			More prone to differentiate into vascular smooth muscle cell		
			Less senescent phenotypes		
			Faster proliferation rate		
			Stronger stemness characteristics		
CD200	Membrane	1st	Much higher level of colony-forming activity	Down-regulated during differentiation	18086871 24721710 28295880 26773707
			Enhanced immune suppression activities	Low level of CD200 expression in MSCs derived from adipose	
			Contributes to immune suppression	Expression is not induced by IFN-γ in AD and UC derived MSCs	
			Contributes to myeloid differentiation inhibition		
			Prone to differentiate into the osteocytes		
Ly6a	Membrane	1st	Contributes to maintain the stem cell state of MSCs	Heterogenous	12732718 18599810 34341173 35445270 17379763
			Higher proliferation rate	Not specific,expressed in multiple types of stem cells	
			Better immune suppression abilities		
THY1	Membrane	1st	Contributes to regulate the balance between osteoblasts and adipocytes differentiation	Not specific,expressed in the fibroblasts	30089635 18985728 26718647 29371677 25739049
			Predict the immune suppressive function		
			Proliferate faster and better differentiation capabilities		
ITGA6	Membrane	1st	Higher colony-forming activities	Not specific,expressed in multiple types of cells	18818395 22311737 23132820 26013602 31494092 33704842 29720266
			Higher differentiation abilities		
			Smaller size		
			Better homing activities		
			Better regeneration activity		
			Better anti-apoptotic activities		
STRO-4	Antibody recognizing the Hsp90β	1st	Higher colony-forming activities		19327008
			Higher proliferation rate		
			Higher multiple differentiation abilities		
ENTPD1	Membrane	1st	Contributes to suppress T cell proliferation		21176405 24043462 28210258 32565049 23804221 31076346
			Contributes to suppress activation of B cells		
			Better efficiency of chondrogenic and osteogenic differentiation		
			Prevent osteoclastogenesis		
CSPG4	Membrane	1st	Enhanced proliferation abilities	Not specific, expressed in fibroblast and endothelial cells	23611563 19462316
			Enhanced colony-forming abilities		
			Enhanced differentiation abilities		
MX1	Intracellular (cytosol, nuclear membrane)	1st	Prone to differentiate into osteoblasts and regenerate the bone		22385654
ITGAV	Membrane	1st	Faster proliferating rate		31747966 33968928
			Better abilities of colony-forming		
			Better homing ability		
			Better anti-inflammatory effects		
			Therapeutic effects in the mouse model of myocardial infarction		
STRO-3	Antibody recognizing the TNSALP	1st	Higher proliferation and differentiation potencies		18158854 19231391 20850099 21155976 22404141 23658436 26799116 28173831 33045417
			Have been applied in treating disease models		
GLI1	Intracellular (nucleoplasm, cytosol)	1st	Responsible for tissue regeneration after injury	Prone to differentiate into osteochondrogenic lineages	24506883 27618218 29230039 33046884 36092701 25799059 25465115 28457748 32783935
				Contributes to tissue fibrosis	
				Heterogenous	
ISLR	Extracellular (secreted)	1st	Predict differentiation efficiency	Expression in fibroblast	26924503 34676218
			Enhanced anti-fibrosis functions		
TNFRSF10D	Membrane	1st		Reduced proliferation rate and differentiation efficiency	28962588
				Increased senescence phenotype	
EPHA7	Membrane	1st	Proliferate faster		31471947
			Higher level of colony-formation		
			Higher level of differentiation capabilities		
SDC2	Membrane	1st	Enhanced colony forming		29979191 32169108 33158246 34746723
			Enhanced immune suppression		
			Enhanced regeneration activities		
			Safe, feasible, and effective in clinics		
ENG	Membrane	1st		Reduced adipogenic differentiation efficiency	20153525 21205995 23069852 33800564
				Contravercy on the osteogenic and chondrogenic differentiation efficiency	
				Not specific,expressed in activated ECs and immune cells	
NGFR	Membrane	1st	Higher colony forming activity		12135677 16977637 17395729 22268519 29482445 29915318 22048731 22982680 30816233 33653407
			Proliferate faster		
			Enhanced immune suppression activity		
			Higher tri-differentiation efficiency		
			Improved therapeutic effects		
NES	Intracellular (intermediate filaments)	1st	A MSC subpopulation constituting the niche for HSCs		20703299 31029167
			Improved therapeutic effects		
FZD9	Membrane	1st	CD349^−^ MSCs have better neovascularization abilities		20658518
CD34	Membrane	1st	Higher colony forming activity	A marker of endothelial cells	1720038 17786605
PDGFRB	Membrane	1st	Contributes to maintain MSC functions		14766732 16210003 17872908
			Higher colony-forming activities		
SSEA-4	Membrane	1st	Enrich MSC populations		17062733
NCAM1	Membrane	1st	Enhanced chondrogenic differentiation potentials	Not specific	19066333 30676001
			Supporting long-term hematopoiesis		
VCAM1	Membrane	1st	Contributes to immune suppression function of MSCs	Colony-forming ability varies among different MSCs	20130212 23555021 32597552 24052950 27044487 35768999
			Stronger ability to suppress immune responses	Expression level decreases during expansion	
			Enhanced homing capacity	CD106 expression varies among MSCs derived from different tissues	
			Better pro-angiogenic activity		
LEPR	Membrane	1st	Much higher colony-forming activities		24953181 27053299
			Promotes adipose differentiation of MSCs while inhibit osteogenesis		
PDGFRA	Membrane	1st	Enhanced differentiation activities	Expressed in human skeletal muscle	19841085 23776077 29529192
			Enhanced differentiation activities	Species differences	
SUSD2	Membrane	1st	Higher clonogenicity		22469435
EPHB2	Membrane	1st	Improves intestinal homing abilities		23413357
			Promotes the intestinal stem cell regeneration		
KIT	Membrane	1st	Better cell proliferative		17348807 24713343
			Enhanced clonogenic activities		
EPHA2	Membrane	1st	Distinguish MSCs from fibroblasts	Not specific,expressed in epithelial cells	26700997 30342659 32899389 28171762 32811512
BST2	Membrane	1st	Up-regulated mRNA levels of immunosuppressive genes	The fresh CD317^−^ BM-MSCs have better immune suppression activities	26070611 35734183 unpublished data
			Better regeneration capabilities		
			Better immune suppression capabilities of UC-MSCs in CDM		
TLX1	Intracellular	1st	Better colony forming potentials		27939685 31320650
			Better tri-lineage differentiation		
ROR2	Membrane	1st	Enhanced chondrogenic differentiation efficiency		28833807
CD274	Membrane	1st	Enchanced immune suppression activities	Widely expressed on multiple types of cells, such as lymphocytes	32707035
			Improved therapeutic effects		
ABCG2	Membrane	1st	Higher telomerase activity		21312316
			Better anti-inflammatory effects		
PODXL	Membrane	1st	Higher colony-forming activities		18818395 22311737 23132820 26013602 31494092 33704842
			Higher differentiation abilities		
			Smaller size		
			Better homing activities		
			Better regeneration activity		
			Better anti-apoptotic activities		
S100A9	Extracellular (secreted)	2st	Enhanced wound repair capabilities		36504438
F3	Membrane	2st	Better regeneration abilities	Lower proliferation	32252818 36504438
LRRC75A	Intracellular (nucleoplasm)	2st	Better VEGF production		37263619
SERPINF1	Extracellular (secreted)	2st	Stable		35445270
CMKLR1	Membrane	2st	Stronger immune suppression activities	Slower proliferation rate	35365767
			Better osteogenic differentiation potential	Weaker adipogenic differentiation potentials	
HMMR	Membrane	2st	Related to cell cycle status		31068579
LGR5	Membrane	2st	Promote alveolar differentiation	A marker for epithelial stem cells	26460010 28886383
LGR6	Membrane	2st	Supports the airway differentiation	A marker for epithelial stem cells	26460010 28886383
			Supports the Lgr5 + epithelial stem cells turnover		

There are various strategies available for biomarker discovery, and among them, two classic approaches stand out: the candidate biomarker strategy and the high-throughput screening strategy. The candidate biomarker strategy is based on existing biological knowledge, where one or more molecules or features possibly related to a specific disease or biological process are selected as candidate biomarkers. These candidates are then experimentally validated for their expression levels or variations under different conditions. This strategy relies heavily on a profound comprehensive understanding of medical domains and relevant biological processes [16]. In contrast, the high-throughput screening strategy employs techniques like genomics, transcriptomics, proteomics, lipidomics, and metabolomics to simultaneously analyze a large number of molecules and features. Through these techniques, it becomes feasible to detect thousands of molecules, facilitating the comparison of differences between heterogenous cell populations. Notably, this approach allows for the identification of biomarkers associated with specific functions without being reliant on prior knowledge [16–20]. It's worth noting that these strategies can be combined to enhance the comprehensive development of potential biomarkers. This integrated approach harnesses the biological knowledge of the candidate biomarker strategy while utilizing the technical capabilities of the high-throughput strategy to discover biomarkers associated with distinct functional subgroups in a more comprehensive and precise manner [16, 21–24]. In the realm of MSCs, with the use of prior knowledge and high-throughput technologies such as single-cell RNA sequencing (scRNA-seq), specific markers related to different functional subsets of mesenchymal stem cells can be more comprehensively and accurately mined [17–24].

Therefore, in the current review, we would discuss the MSC markers that have been identified so far. Furthermore, based on the identification approaches, these markers have been categorized into two groups: the 1st generation of MSC markers, which has been identified by the candidate biomarker strategy; and the 2nd generation of MSC makers, which has been identified by high-throughput screening approaches (Table 2).

### Techniques of identifying mesenchymal stem cell subpopulations

In most studies reviewed in this paper, Flow cytometry and fluorescence-activated cell sorting (FACS) is predominantly utilized for sorting MSC subpopulations. FACS are the primary methods for identifying MSC subpopulations, celebrated for their precision and versatility in scientific research. These technologies use fluorescently labeled antibodies to target specific surface markers, allowing researchers to conduct multiparameter analyses [25, 26]. This facilitates simultaneous assessment of various markers and functional properties within MSC populations, aiding in the identification and isolation of distinct subpopulations based on differential expression of markers. Such detailed analysis provides crucial insights into MSC heterogeneity.

Another vital technique, immunomagnetic cell sorting, utilizes magnetic beads tagged with antibodies targeting specific surface markers for selective isolation of MSC subpopulations [27, 28]. This method ensures high specificity and efficiency, essential for distinguishing and harvesting functionally diverse MSC subsets.

Additionally, functional assays are integral for understanding the biological characteristics of MSC subpopulations. Immunomodulatory assays, for instance, involve co-culture setups with immune cells to evaluate MSCs' effects on immune cell proliferation, activation, and cytokine production [29–33]. These studies highlight the potential therapeutic uses of distinct MSC subsets in treating immune-related conditions. Differentiation assays, including those for osteogenic, adipogenic, and chondrogenic pathways, further elucidate the multilineage potential of MSC subpopulations, critical for identifying suitable cell sources for tissue engineering and regenerative medicine.

Gene expression profiling, through techniques such as RNA sequencing, provides deep insights into the transcriptomic landscapes that define specific functional states or lineage commitments within MSC populations [17–19, 23, 34]. These analyses help pinpoint molecular signatures characteristic of unique MSC subsets, enhancing our understanding of their heterogeneity.

Together, these techniques not only facilitate a comprehensive analysis of MSC heterogeneity but also specialize in pinpointing distinct MSC subpopulations. By employing these advanced methodologies, researchers can effectively characterize the diverse functional capacities and biological properties inherent to each subpopulation, significantly enhancing the precision of mesenchymal stem cell-based therapeutic strategies and the development of personalized regenerative medicine.

## 1st generation of MSC markers

### Immune suppression related markers

Although the MSCs have been widely investigated in the animal models of different diseases, the only approved clinical product of MSCs is for GVHD (Graft Versus Host Disease) treatment in clinics [35, 36], because of their immune suppression capabilities. The immune modulation activity is one of those important contributors to the therapeutic effects of MSCs [1].

#### Extracellular secreted modulators

It has been demonstrated that TNFAIP6 (Tumor Necrosis Factor Alpha-Induced Protein 6) is a potential cell marker for mouse MSCs, irrespective of tissue origin and laboratory origin, with higher immune suppression activities and improved therapeutic effects [12]. However, the membrane expression level of TNFAIP6 is significantly lower than its cytoplasm level [12]. Indeed, TNFAIP6, also known as TSG6, is a small secreted protein with extracellular matrix remodeling and immunomodulation functions [37]. On the other hand, the importance of these secreted modulators, such as the TNFAIP6 having been characterized as one efficacy predictor of MSCs in treating inflammation in vivo [38], makes it necessary to develop novel strategies to purifying these MSC subpopulations for improving their therapeutic effects.

#### Extracellular ATP clearance

Dying or stressed cells could release ATP (Adenosine 5'-triphosphate) to the extracellular spaces and induce the pro-inflammatory cascade [39, 40]. The immune regulatory cells, such as Treg and MSCs, could express genes, such as ENTPD1 (Ecto-Nucleoside Triphosphate Diphosphohydrolase 1, also known as CD39) and NT5E (Ecto-5′-AMP-nucleotidase, also known as CD73), responsible for clearing these extracellular ATP [39, 41]. CD39 could hydrolyze the extracellular ATP, into ADP and then AMP; while CD73 converts AMP into adenosine [39, 40]. The extracellular adenosine has strong immune suppression activities via binding to the corresponding P1 receptors (including A1R, A2_A_R, A2_B_R, and A3R), and activating the downstream pathways (such as PKA, NF-κB, CREB, AKT, PI3K, ERK, JNK, and p38) [42]. Furthermore**,** the extracellular adenosine also regulates other cell functions, such as cell proliferation, adhesion, migration, invasion, tight junction formation, and vascular remodeling [39, 40, 42, 43].

The expression levels of CD39, CD73, and adenosine receptors could be induced by tissue damage, remodeling, and also the conditions of hypoxia and inflammation [41–43]. It has been demonstrated that MSCs express both CD73 and CD39 and could convert ATP into adenosine, resulting in suppressing T cell proliferation [44–47], and the activation of B cells [48]. The expression levels of CD73 modulate the proliferation and differentiation capabilities of MSCs [49, 50]. Its expression level decreases during the differentiation process [51].

The purified CD73^+^ MSCs have higher levels of colony-forming capabilities [52], even higher than the ENG^+^ and THY1^+^ MSCs [51]. In addition, CD73^+^ MSCs have much higher tri-differentiation abilities (adipocytes, osteoblasts, and chondrocytes) and higher immune suppression activities [52, 53]. Through EGFP reporter analysis in mice, CD73 could identify the MSCs in different organs in vivo [54, 55]. Furthermore, CD73^+^ MSCs are much more smaller with spindle and rod-like shapes, while CD73^−^ MSCs are more polygonal larger cells [33]. CD73^+^ MSCs secrete higher levels of regeneration cytokines, such as VEGF, SDF-1α, and HGF than CD73^−^ MSCs, and show improved therapeutic effects on the rat model of myocardial infarction [33]. Furthermore, CD73^+^CD39^+^ MSCs have great potential in bone regeneration, including better efficiency in chondrogenic and osteogenic differentiation [56], preventing osteoclastogenesis [57], and promoting bone formation via the Wnt/β-catenin pathway[58].

#### Other immune regulators

CD200 is an immune suppressor and promotes peripheral immune tolerance [59, 60]. Its immune suppression function works through binding to its receptor CD200R, which then activates multiple pathways, such as MAPK-ERK, p38 MAPK, and JNK, via Dok and p120-RasGAP [61], resulting in upregulating the downstream effectors including IDO (indoleamine-2,3-dioxygenase), TGF-β, and IL-10 [59]. A higher expression level of CD200 in MSCs correlates with enhanced immune suppression activities in vitro and in vivo [62]. CD200 expressed on MSCs recognizes and binds to its receptor CD200R, which is expressed on myeloid progenitors, resulting in myeloid differentiation inhibition and immune suppression [63]. CD200^+^ MSCs have much higher levels of colony-forming activity [51]. However, it has been demonstrated that the expression of CD200 is undetectable in MSCs derived from umbilical cord blood [64], or very low in MSCs derived from adipose [65]. In contrast, MSCs derived from the umbilical cord express higher levels of CD200 [65]. Interestingly, the pro-inflammatory cytokine IFN-γ upregulates the expression of CD200 in MSCs derived from bone marrow but not adipose or umbilical cord [65].

BST2 (bone marrow stromal cell antigen 2), also known as CD317, is a type of transmembrane glycoprotein involved in virus reproduction suppression and immune regulation [66]. Using the hTERT immortalized human bone marrow MSC colonies, it has been demonstrated that the MSCs from the CD317^+^ colony have increased cell areas and up-regulated mRNA levels of immunosuppressive genes than the CD317^−^ MSCs in vitro [67]. Furthermore, CD317^+^ bone marrow-derived MSCs have better regeneration capabilities than the CD317^−^ MSCs [68]. However, fresh CD317^−^ MSCs isolated from human bone marrow have better immune suppression activities but not CD317^+^ MSCs [68]. However, our unpublished data show that CD317^+^ MSCs isolated from the human umbilical cord and expanded with chemically defined media have better immune suppression capabilities (unpublished data).

CD274, also known as PD-L1 (programmed death ligand 1), is a type I transmembrane protein and is widely expressed on multiple types of cells, such as lymphocytes [69]. Its expression can be induced by pro-inflammatory cytokines, such as interferon-γ (IFN-γ), TNF-α, and IL-17 [69, 70]. And it has strong immune suppression activities through binding to its receptor PD-1 [71]. It has been demonstrated that PD-L1 is expressed in MSCs [70, 72, 73]. PD-L1^+^ MSCs have enhanced immune suppression activities and improved therapeutic effects on the collagen-induced mouse model of arthritis [74].

MX1, for ‘myxovirus resistance’, is the gene responsible for virus immunity and an important component of interferon pathway [75]**.** It has been demonstrated that Mx1^+^ MSCs are clonogenic at the single-cell level and have tri-differentiation abilities [32]. Although its antivirus mechanism remains unsolved, the Mx1^+^ MSCs might also have immune regulatory functions.

### Cell adhesion related markers

In addition to the important role of MSCs in modulating immune responses [1], another critical function is regulating cell adhesion, including both the cell adhesion and migration of MSCs, as well as the recruitment and adhesion of other types of cells, such as lymphocytes.

#### Mediating cell migration

CD44 is an important adhesion molecule involved in recruiting immune cells or stem cells into the inflammatory or injured tissues, via interacting with hyaluronic acid (HA), which is expressed in the injured/inflammatory sites [76, 77].Their interactions induce conformational changes of CD44, recruit adaptor proteins, and lead to cytoskeletal rearrangement, resulting in the activation of various signaling pathways that involve cell growth, adhesion, and migration [76, 77]. In addition, CD44 also functions as a co-receptor to regulate the activities of other receptors, such as VEGFR, EGFR, FGFR and PDGFR [78]. CD44 is widely expressed in multiple types of cells, including MSCs, and it also contributes to MSC recruitment [79, 80]. Its expression level is further induced by PDGF [79]. The migration and adhesion of MSCs depend on CD44-HA (hyaluronic acid) interaction [79, 80]**.** Therefore, CD44 is a potentially important cell surface marker for MSC purification [81]. However, later investigations indicate that freshly isolated mouse/human MSCs derived from bone marrow express very low levels of CD44 [82, 83]. MSCs show enrichment in the CD44^−^ fractions, as evidenced by their marker expression, colony-forming capacity, and in vitro differentiation abilities [82, 83]. Interestingly, CD44 is gradually up-regulated during cell expansion, even for the CD44^−^ fractions of MSCs [82, 83]. Thus, the CD44 expression levels after in vitro expansion, may not reflect their original cell identity [82]. The CD44^+^ MSCs have enhanced colony-forming capacity and differentiation abilities [84].

MCAM (melanoma cell adhesion molecule), also known as CD146, is involved in cell-ECM (extracellular matrix) interactions [85, 86]. Upregulation of CD146 could switch cell–cell adhesion to cell-ECM adhesion by interacting with its ligands in the ECM, preparing cells for migration and invasion by secreting related cytokines and proteins [85, 86]. CD146 is expressed in many cell types, especially in those cells constituting blood vessels, such as endothelial cells [86] and MSCs [87–91]. And it has been proposed that CD146 is an MSC marker of multipotency [90–93]. CD146^+^ MSCs have a much stronger chemotactic attraction [94–97], and enhanced immune suppression activities in vitro and in vivo [27, 97–99]. Higher levels of CD146 expression correlate with a faster proliferation rate, enhanced multilineage differentiation potentials, stronger stemness characteristics, and less senescent phenotypes [98–100]. However, Tormin et al. have demonstrated that the colony-forming cells are exclusively enriched in the CD271^+^ population of MSCs in human bone marrow, regardless of the expression level of CD146 [101]. Within the CD271^+^ MSCs, both CD146^+^ and CD146^−^ share similar genotypes and phenotypes [101]. Furthermore, other studies have also demonstrated that CD146^+^ and CD146^−^ share similar levels of MSC marker expression, colony-forming, proliferation and differentiation capabilities [94, 96, 102, 103]. And the CD146^−^ MSCs even proliferate significantly faster than the CD146^+^ population [103]. Higher expression of CD146 also indicates more prone to differentiate into vascular smooth muscle cells [103]. In MSCs derived from human dental cysts, CD146^Low^ MSCs have higher levels of cell proliferation, colony-formation, and osteogenesis [102].

SDC2 (Syndecan-2), also known as CD362, is a type of transmembrane heparan sulfate proteoglycan, involved in modulating cell adhesion, proliferation, migration, and apoptosis through its interactions with the extracellular matrix and various proteins, such as proteases and cytokines. These interactions induce downstream pathway activations through intracellular protein partners [104]. CD362 is mainly expressed in MSCs [104]. CD362^+^ MSCs have enhanced colony forming, immune suppression and regeneration activities [105–107]. Furthermore, both Phase 1 and Phase 2 clinical studies show that CD362^+^ MSCs are safe, feasible, and effective in treating COVID-19 infections [108].

#### Mediating lymphocyte adhesion

VCAM1 (vascular cell adhesion molecule 1), also known as CD106, mediates cell–cell adhesion and plays an important role in mediating the rolling, adhesion, and migration of circulating lymphocytes on the endothelium under inflammatory conditions [109–111]. The CD106 is induced by pro-inflammatory cytokines in MSCs [112], and is involved in the immune suppression function of MSCs [113]. CD106^+^ MSCs derived from placenta and umbilical cord have stronger abilities to suppress immune responses [112, 114] and better pro-angiogenic activity, with enhanced promoting endothelial cell proliferation and migration [28, 115]. Furthermore, CD106^+^ MSCs have enhanced homing capacity [28, 112].

ITGA1 (integrin subunit alpha 1) is identified in the very late stage of activated T cells. ITGA1 is the major component of the ECM by binding to collagens (mainly collagen I and IV) and laminin, supporting the migration and activation of leukocytes, such as T cells, NK cells, NKT cells, and monocytes, especially the long-term activated or resident T cells [116, 117]. The ITGA1 has been proposed as an MSC marker for human bone marrow [118–120]. The ITGA1^+^ MSCs have stronger colony-forming activity [118, 119]**.**

CD9, also known as MRP1 (motility related protein-1), is widely expressed in many cell types, including MSCs and lymphocytes, and is involved in regulating cell migration and invasion through integrin receptors [121, 122]. It has been demonstrated that CD9 is involved in the recognition and binding between MSCs and lymphocytes [123]. CD9^+^ human MSCs have higher NOS (nitric oxide synthase) expression, proliferation rate, colony formation ratio, and stronger cell adhesion capability, resulting in better engraftment and improved therapeutic effects in the mouse model of hindlimb ischemia [124, 125].

#### Other adhesion molecules

THY1, also known as CD90, is a small membrane protein located in the lipid raft [126]. Although CD90 does not have an intracellular domain, it is involved in cell adhesion, migration, proliferation, and apoptosis through modulating the cell–cell and cell–matrix interactions via binding to its ligands, such as integrins, syndecan, CD90 and CD97 [126, 127]. CD90 has been identified as an important marker for MSCs from different species and tissues [128–132], and could be a potential marker for predicting the immune suppressive function of MSCs [133, 134]. Later studies also indicate that CD90^+^ MSCs have a faster proliferation rate and better differentiation capabilities [135–137]. However, CD90 is also expressed in the fibroblasts, which might induce fibrosis [138].

Other adhesion genes also have been demonstrated as potential MSC makers, such as the SUSD2 [139–141], ALCAM [142, 143], NCAM1 [144–149], CD51 (also known as ITGAV) [150, 151], and ITGA6 (also known as CD49f) [152] (Tables 1, 2).

### Regeneration related markers

#### Ephrin receptors

The Ephrin receptors (EphA and EphB), which can be recognized by ephrin ligands, play an important role in modulating multiple cellular functions, such as the self-renewal and differentiation of stem cells [153–158]. Proteomics studies indicate that EphA2 is expressed in the MSCs from human bone marrow and umbilical cord, and regulates the functions of MSCs [159, 160]. Follow-up studies showed that EphA2 could be a cell surface marker to distinguish MSCs from fibroblasts [161]. Furthermore, EphA7^+^ MSCs proliferate faster and have higher levels of colony formation and differentiation capabilities [162]. And EphB2^+^ MSCs have improved intestinal homing abilities and promoted the intestinal stem cell regeneration [31]. It has been demonstrated that Eph/ephrin pathway is also involved in the cell migration of MSCs [163–166], and is essential for suppressing the proliferation of activated T cells by MSCs [167].

#### PDGFR

PDGFR (platelet-derived growth factor receptor), including PDGFRA and PDGFRB (also known as CD140α and CD140β, respectively), plays an important role in embryonic development and organogenesis, particularly in regulating the proliferation, migration, and differentiation of MSCs in various organs [168–171]. Although both CD140α and CD140β have been identified as MSC markers [91, 168, 169], their investigation also indicates that CD140α is the negative selection marker for human MSCs derived from bone marrow, which differs from mouse MSCs [172].

#### Wnt pathway

FZD9, also known as CD349, is a receptor for Wnt ligands and activates β-catenin signaling pathway, which is involved in embryonic development and stem cell renewal [173, 174]. It has been demonstrated that CD349 is expressed in MSCs from both bone marrow and placenta, and proposed as a feasible marker for MSC isolation [175, 176]. Although both CD349^+^ and CD349^−^ MSCs show similar levels of MSC marker expression and differentiation abilities, the CD349^−^ MSCs have better neovascularization abilities than the CD349^+^ MSCs [177].

ROR2 is a tyrosine kinase-like orphan receptor, which can be activated by Wnt5a and regulate the tissue polarity and cell movement through downstream WNT/PCP (planar cell polarity) signaling pathway [178, 179]. It has been demonstrated that ROR2^+^ MSCs derived from human bone marrow have enhanced chondrogenic differentiation efficiency [30].

#### Others

ALDH (aldehyde dehydrogenase) belongs to the metabolic enzyme family, which is involved in regulating glycolysis/gluconeogenesis and the detoxification of aldehydes via oxidation [180, 181]. It plays an important role in cell survival, proliferation, differentiation, and has been characterized as a classical stem cell marker [180, 181]. In human adipose tissues, the ALDH^High^ MSCs represent a more primitive subpopulation than the ALDH^Low^ MSCs, from the perspectives of cell proliferation and tri-differentiation capabilities [182, 183].

STRO-1 can bind to an uncharacterized cell surface antigen, and identify around 10% of mononuclear cells in the human bone marrow [184]. Purified STRO-1^+^ cells from human bone marrow have higher levels of colony-forming activity, proliferative rate, multilineage differentiation capabilities, and immune suppression activities by expressing higher levels of immune inhibitory factors (IL-8, LIF, IDO, HLA-G, VCAM1, TGF-β, and IL-10) [185], suggesting that STRO-1 is a potential MSC marker [184, 186]. Later study showed that STRO-1^+^ MSCs have better homing activities than STRO-1^−^ MSCs in the bone marrow, spleen, muscle, liver and kidney, while STRO-1^−^ MSCs are more prone to be trapped in the lung [187].

STRO-3, which recognizes TNSALP (tissue nonspecific alkaline phosphatase, a cell-surface glycoprotein), also identifies a MSC subpopulation with higher proliferation and differentiation potencies [188, 189]. The STRO-3^+^ MSCs have been identified in various species and tissues and applied in treating various disease models [189–196].

STRO-4 is a monoclonal antibody recognizing the cell surface expressed chaperone protein, Hsp90β. STRO-4^+^ MSCs have higher colony-forming activities, proliferation rates, and multiple differentiation abilities [29].

TLX1, also known as Hoxa11, belongs to Hox gene family which is essential for patterning during embryonic development. It has been demonstrated that the *Hoxa11*-lineage marked (Hoxa11-eGFP) could identify the multi-potent MSCs in the mouse bone marrow [197]. Hoxa11^+^ MSCs have better colony forming potentials and tri-lineage differentiation abilities [198, 199].

Transcription factor GLI1, the effector of the Hh signaling pathway, which regulates tissue development and homeostasis, has been used to mark MSCs in vivo [24, 200–202]. These Gli1^+^ MSCs are responsible for tissue regeneration after injury [200, 203–206]. However, the Gli1^+^ MSCs have the tendency to differentiate into osteochondrogenic lineages [201, 204]. Furthermore, the Gli1^+^ MSCs also contribute to tissue fibrosis [205, 207, 208].

ISLR, also known as Meflin, is a glycoprotein (cell membrane located or secreted) with anti-fibrosis functions through interacting with BMP7 (bone morphogenetic protein 7) and inhibiting TGF-β pathway and myofibroblast differentiation [209]. It has been demonstrated that Meflin is one MSC marker, and its expression positively correlates with its differentiation efficiency [210, 211].

Sca-1 (stem cell antigen-1) has been characterized as a common marker in multiple types of stem cells, such as hematopoietic stem cells and MSCs [212, 213]**.** It has been demonstrated that mouse MSCs derived from bone marrow and ear express high levels of Sca-1 [214–216]. The expression of Sca-1 is fundamental for maintaining the stem cell state of MSCs [22, 213, 215, 217]. Furthermore, they have higher proliferation rates and better immune suppression abilities [22, 217]. Other common stem cell markers, such as SSEA-4 (stage-specific embryonic antigen-4), KIT, and ABCG2, have also been identified as MSC markers [218–227].

### Neuron related markers

Interestingly, the MSCs express some neural development related genes and some of them have been identified as MSC markers, such as CSPG4 (chondroitin sulfate proteoglycan 4) [228–230], GD2 (Disialoganglioside) [231], CD271 [232–238], and Nestin [239–243]. Whether the expression of neuron related genes indicates the dedifferentiated state of MSCs or potential interactions between MSCs and neurons remains unclear and needs further investigation.

### Other markers

Since the first demonstration of MSCs, the ENG (Endoglin), also known as CD105, has been identified as a classical MSC marker [15, 128]. Using CD105 to purify MSCs is feasible and efficient in human bone marrow and adipose [244–248]. Furthermore, CD105^+^ MSCs have increased osteogenic and chondrogenic differentiation efficiency, and reduced adipogenic differentiation efficiency [248, 249]. However, controversial results also show that a low expression level of CD105 is correlated with increased osteogenic and chondrogenic differentiation [250]. Indeed, as a coreceptor of the TGF-β superfamily, CD105 is involved in regulating osteogenic differentiation [251–253].

The SDF1-CXCR4 is the major pathway responsible for cell recruitment and retention [254, 255]. CXCR4 is expressed in human MSCs and contributes to the MSCs homing process [256–260]. For example, in the mouse model of osteogenesis imperfecta, the human MSCs migrate into the bone marrow through the SDF1-CXCR4 pathway and reduce the fracture rate [261]. Furthermore, in the rat model of ischemic brain injuries, rat MSCs migrate into the injured sites of the brain and show therapeutic effects via the SDF1-CXCR4 pathway [262, 263]. Although the expression level of CXCR4 is high in MSCs, few the on the cell surface [256, 261]. However, Honczarenko et al. have demonstrated that the surface expression of CXCR4 is up to around 43% [264], indicating that some factors might induce the cell surface expression of CXCR4, such as culture conditions, stimuli, and passage numbers [265]. Indeed, the expression level of CXCR4 decreases during passaging [264, 266] and aging [267]. The cell membrane localization of CXCR4 is induced by cytokine stimulation (such as SDF-1) [256, 261, 268] or 3D culture conditions [269].

Some other MSC markers have also been demonstrated, such as LepR (Leptin receptor) [270–273], CD34 [274–276], and TNFRSF10D [277]. However, their functions in MSCs remain unclear. Purifying MSC subpopulation with one single maker has many disadvantages (Table 2). Therefore, the combination of multiple markers is a promising strategy to improve the efficiency and efficacy of MSC subpopulation purification. It has been demonstrated that the PODXL^hi^/ITGA6^hi^ MSCs have better activities of colony formation, differentiation, proliferation, homing activities, regeneration activity, and anti-apoptotic activities [152, 278–282]. The PDGFR^+^Sca-1^+^ MSCs could differentiate into both mesenchymal and endothelial at single-cell level with enhanced self-renewal and multipotency abilities [129], and the CD146^+^PDGFRβ^+^ MSCs have higher levels of colony-forming activities [91]. Combining PDGFRα and other markers, such as Ly6a, Sca-1, and CD51, would further enrich the MSC subpopulation with enhanced colony-forming and differentiation activities [129, 243, 283, 284]. On the other hand, identifying novel MSC markers with novel high-throughput technologies is also critical for both MSC subpopulation purification and understanding the heterogeneity of MSCs.

## 2nd generation of MSC maker identification-high-throughput approach

The emergence and development of high-throughput technologies (genomics, transcriptomics, proteomics, lipidomics, metabolomics, and so on) have revolutionized various fields of life sciences [285–287]. These high-throughput technologies have not only expedited the pace of research but also transformed our understanding of life itself by providing a comprehensive and intricate view of biological systems. Since the introduction of Illumina's Solexa sequencing technology in 2005, a new era has been heralded by paving the way for high-throughput technologies [288]. This pioneering approach, built upon parallel sequencing principles, enables the simultaneous analysis of millions of DNA fragments, dramatically boosting sequencing efficiency. Notably, this breakthrough laid the foundation for subsequent advancements, with other platforms such as 454 Life Sciences, Ion Torrent, and PacBio also contributing to the progress of high-throughput technologies [285].

The impact of high-throughput technology extends far beyond genomics, reverberating profoundly across various domains of omics research. In the realm of transcriptomics, we can now unravel intricate gene regulatory networks by simultaneously analyzing the expression of thousands of genes. Technologies like RNA-Seq have empowered scientists to assess gene expression patterns across different conditions, tissues, or developmental stages, shedding light on cellular processes and signaling pathways [286]. High-throughput mass spectrometry techniques in proteomics offer a swift and comprehensive understanding of protein–protein interactions, modifications, and functions within cells. These methods allow researchers to identify and quantify proteins in complex samples, revealing insights into cellular processes, biomarker discovery, and disease mechanisms [287]. In lipidomics, mass spectrometry-based methods have enabled the comprehensive analysis of lipid molecules in biological samples, uncovering lipid profiles associated with health and disease [289]. Similarly, metabolomics, utilizing high-throughput mass spectrometry and nuclear magnetic resonance (NMR) techniques, offers insights into the global metabolite composition of cells or organisms, contributing to our understanding of metabolic pathways and disease biomarkers [290]. Epigenomics, focusing on epigenetic modifications like DNA methylation and histone modifications, benefits from high-throughput techniques such as DNA methylation arrays and next-generation sequencing. These tools provide a genome-wide view of epigenetic modifications, aiding in deciphering their roles in gene regulation, development, and disease [291].

Among these high-throughput technologies, single-cell RNA sequencing technology (scRNA-seq) is a significant innovation in the field of MSCs that has sparked widespread interest in recent years [17–24]. By deciphering the gene expression of each individual cell within a cell population, this technique reveals the astonishing complexity of cellular diversity and heterogeneity, bringing about a revolutionary breakthrough in cellular biology research [292, 293]. Distinct from traditional bulk RNA sequencing methods, scRNA-seq can precisely analyze cell function and types, regardless of sample heterogeneity [293–295].

Several novel MSC markers have been discovered since the application of scRNA-seq technology in the MSC field, such as the LRRC75A^+^ MSCs with enhanced VEGF production [23]; the CMKLR1^+^ MSCs with improved immune suppression capabilities [19]; the F3^+^ and S100A9^+^ MSCs with better regenerative activities [17, 18, 34].

Among these novel MSC markers identified by scRNA-seq, the CMKLR1^+^ subpopulation with enhanced immune suppression capabilities [19] has been investigated in detail. The CMKLR1 (Chemokine-like receptor 1), also known as CCRL2 (chemokine C–C motif receptor-like 2), is the transmembrane receptor for chemoattractant chemerin, involved in recruiting and migrating of lymphocytes and immune suppression via its ligand resolvin E1, an important anti-inflammatory mediator [296]. Furthermore, it has been demonstrated that the CMKLR1^+^ MSCs have better osteogenic differentiation potential and weaker adipogenic differentiation potentials than the CMKLR1^−^ MSCs [19]. Indeed, the CMKLR1 pathway regulates the differentiation balance between the osteoblastogenic and adipogenic MSCs [297]. However, their data also indicate that inhibiting the CMKLR1 pathway promotes the osteoblastogenic differentiation of MSCs and suppresses the adipogenic differentiation of the mouse MSCs [297]. Whether the controversial data resulting from species differences needs further investigation [298–300].

The applications of scRNA-seq not only promote the identification of novel MSC markers, but also uncover new potential functions of MSCs. The MSC marker F3 [17, 18], also known as CD142 or thromboplastin, is a transmembrane glycoprotein and a receptor for coagulation factors, which is involved in platelet activation and coagulation development after tissue injury [301, 302]. The discovery of F3 in MSCs might indicate that MSCs play an important role in blood clot formation at the site of injury.

The extracellular matrix modification function of MSCs is well-known [303, 304]. However, the extracellular matrix microenvironment is a highly complex and dynamic biological component and is critical for the functions of MSCs, including the immune modulation function and stem cell characteristics [3, 305, 306]. Identifying new extracellular matrix-related MSC markers, such as Serpinf1 [22] and HMMR [20], would enhance our understanding of MSC biology in greater depth and breadth..

In addition, scRNA-seq is a powerful strategy for investigating the heterogeneity of MSCs. Purifying a homogenous MSC subpopulation is proposed to have improved therapeutic advantages [17, 19, 23, 307]. However, it has been demonstrated that the Gli1^+^ MSCs are still heterogenous, as revealed by scRNA-seq [24]. Furthermore, scRNA-seq can also uncover the diversity of functions and interactions among different MSC subpopulations. Two major MSC subpopulations (Lgr5^+^ and Lgr6^+^) residing in the mouse lung have completely different functions, uncovered by scRNA-seq analysis [21]. Lgr6^+^ MSCs support the airway differentiation, while the Lgr5^+^ MSCs promote alveolar differentiation [21]. In the human umbilical cord, four different MSC subpopulations (proliferative, niche-supporting, metabolism-related, and biofunctional MSCs) have been revealed by scRNA-sequencing [17].

### High-throughput techniques for purifying MSC subpopulations

Throughout the developmental trajectory of scRNA-seq, various innovative platforms have emerged, each catering to different research needs based on their unique principles and features. The 10 × Genomics Chromium system is one widely used platform. It employs droplet technology to combine individual cells with specific molecular barcode particles, enabling high-throughput cell capture and transcriptome sequencing [308]. Similarly, Drop-seq utilizes droplet technology to encapsulate cells and molecular barcode beads in droplets, providing a cost-effective option for large-scale cell sequencing [308]. For studies requiring more accurate and comprehensive gene expression information, SMART-seq2 is an ideal choice. Its principle involves introducing specific sample labels after reverse transcription of RNA, allowing individual processing and sequencing of each cell's RNA for deeper insights [309]. For large-scale sample processing, CEL-seq2 proves to be a powerful selection, utilizing cell-specific molecular barcode primers to provide unique identification for each cell [310]. Additionally, the C1 platform, also known as Fluidigm C1, is an advanced single-cell analysis technology platform. It combines microfluidics technology and real-time fluorescence PCR technology, enabling high-throughput capture, processing, and analysis of individual cells. The workflow of the C1 platform includes key steps such as cell capture, lysis, reverse transcription, and amplification, resulting in high-quality single-cell transcriptome data. The C1 platform can be applied to various types of cell analysis, offering crucial support for cellular biology research [311].

Apart from the aforementioned platforms, other unique single-cell sequencing platforms continue to advance the field of cell analysis. For instance, inDrop, a platform similar to Drop-seq, utilizes microfluidic chips for cell capture, boasting high-throughput performance. Its distinctive design involves encapsulating cells and molecular barcode beads together in droplets, enhancing efficiency and accuracy in cell capture and analysis [308]. Moreover, sci-ATAC-seq is another notable platform that not only focuses on single-cell gene expression but also integrates transcriptome and chromatin accessibility information, providing researchers with more comprehensive data [312]. SPLiT-seq, a high-throughput single-cell sequencing technology, simultaneously captures the transcriptomes of thousands of cells. Through specialized fragmentation tags, cellular lysates are split into multiple fragments, each containing a cell-specific molecular barcode. This tag design enables the concurrent amplification of RNA fragments from multiple cells in a single reaction, achieving high-throughput cell capture and sequencing [313].

The exploration of the majority of these markers (S100A9, F3, LRRC75A, SERPINF1, CMKLR1, GL1) in the context of scRNA-seq applications has primarily relied on the 10 × Genomics Chromium system [17–19, 22–24], while the CD168 identification was conducted with the C1 platform [20] and the interaction between Lgr5^+^ and Lgr6^+^ MSCs were carried out by using the SMART-seq2 technology [21]. The reliability of these three platforms for developing novel MSC markers has been successfully validated. However, other single-cell sequencing platforms have yet to be applied in the MSC marker field so far. Their respective unique advantages, however, suggest they still hold immense potential for the development of new and effective MSC markers.

Beyond accelerating the pace of analysis, these technologies facilitate the simultaneous analysis of expansive datasets, laying bare the intricate network of molecular mechanisms and relationships that drive biological systems [285]. With the wide application of high-throughput technologies in biomedicine, we also had a deeper understanding of the complexity of biological systems and sought to go beyond the limitations of single omics. The rise of high-throughput technologies not only accelerated data generation but also paved the way for the emergence of multi-omics. This approach, fueled by the copious data generated, marries different omics layers (genomics, transcriptomics, proteomics, and metabolomics) into a comprehensive narrative of biological intricacies. By merging diverse omics datasets, multi-omics integration offers a more comprehensive biological context, enhancing accuracy and facilitating meaningful interpretation of findings [314, 315]. Notably, multi-omics technologies have already been applicated in MSCs [316–319]. Gao et al. demonstrated the utility of multi-omics analysis in understanding the immunosuppressive efficacy of MSCs, shedding light on cellular senescence and PD-L1 expression through single-cell transcriptome and proteomic data analysis [319]. Their findings underscore the potential of multi-omics approaches in discovering new effective MSC markers. This indicates that multi-omics is a feasible strategy to find new effective MSC subpopulations.

### Enhanced therapeutic efficacy of marker-sorted MSC subpopulations

Above-mentioned MSC subpopulations exhibit enhanced therapeutic efficacy in various disease models, offering tailored treatment approaches for regenerative medicine and immunotherapy. CD73^+^ MSCs, characterized by heightened regeneration cytokine secretion and colony-forming capabilities, have shown promising results, particularly in myocardial infarction models [33, 52]. Similarly, CD200^+^, CD317^+^, and PD-L1^+^ MSC subpopulations demonstrate superior colony-forming activity and immune modulation, with CD317^+^ MSCs exhibiting notable immune suppression capabilities [51, 68, 74]. Moreover, functional diversity is evident among MSC subpopulations. For instance, CD146^+^ MSCs exhibit strong chemotactic attraction and immune suppression, while CD362^+^ MSCs display enhanced colony formation and immune suppression, validated in COVID-19 clinical trials [27, 94–99, 105–108]. Additionally, CD106^+^ MSCs from placenta and umbilical cord tissues demonstrate potent immunomodulation and pro-angiogenic activities [28, 112, 114, 115]. Furthermore, ITGA1^+^ MSCs and STRO-4^+^ MSCs exhibit robust colony-forming and proliferation rates [29, 118, 119].

In the clinical translation of marker-based sorting, personalized therapeutic interventions are becoming increasingly feasible. However, further clinical studies are needed to validate the efficacy and safety of these approaches. Overall, leveraging the unique properties of marker-sorted MSC subpopulations holds great promise for advancing regenerative medicine and immunotherapy, offering tailored treatments for diverse medical conditions.

## Conclusions and perspectives

Although the therapeutic applications of the MSCs have great promises, challenges still need to be overcome [320, 321]. And the heterogeneity of MSCs constitutes one of those important barriers before their clinical application [6, 7]. Through bioinformatic analysis of the RNA-seq data from different labs and tissues, it is shown that the isolation and expansion procedures induce more heterogeneity than the tissue origin [12]. It should be noted that purifying and expanding the MSCs in vitro is a kind of stress similar to tissue damage in vivo, which might affect the molecular pathways and functions of MSCs [322]. Indeed, the expanded MSCs in vitro are very different from their counterpart in vivo [323]. The MSC expansion strategy would select the cell population which could adapt to these stimuli and stresses [322], indicating the necessity of standardizing the MSC processing procedures and developing a full chemical defined medium [6, 10–12]. Therefore, selecting the suitable MSC subpopulations with specific markers based on their functions and applications is necessary and mandatory [6, 7].

So far, the quest for identifying markers of MSCs has been incessant. The emergence of advanced high-throughput multi-omics techniques offers a promising avenue for discovering novel markers. In this review, numerous MSC subpopulations identified through marker-based sorting have demonstrated significant therapeutic efficacy in animal models. These subpopulations mainly exhibit enhanced therapeutic effects through their potent immunosuppressive capabilities, which have been validated across various animal models of inflammation [38, 68, 74, 112, 114]. Additionally, some subpopulations possess superior homing and regenerative properties, contributing to tissue repair in the rat model of myocardial infarction and the mouse model of hindlimb ischemia [31, 33, 124, 125]. These findings offer promising directions for future therapeutic applications of MSCs. Furthermore, ongoing advances in understanding and manipulating the properties of MSC subpopulations hold great promise for the development of more targeted and effective therapies in regenerative medicine and immune modulation.

However, upon evaluating the majority of currently developed markers, a trend becomes apparent: many subpopulations that are sorted tend to revert to an unsorted state after multiple generations of in vitro proliferation. For instance, during the isolation of MSCs using markers like MCAM, CD9, CXCR4, and STRO-1, their expression diminishes upon subsequent in vitro expansion and cultivation [100, 124, 264, 266, 324]. This situation might indicate that the sole reliance on biomarkers cannot purify consistent and stable subpopulations of MSCs, and eventually achieve successful applications in clinical medicine.

Under diverse physiological or pathological conditions, MSCs exhibit various forms of plasticity, including alterations in morphology, surface markers, secretion, differentiation, proliferation, migration, and apoptotic potential [325]. This plasticity is intimately linked to the microenvironment surrounding MSCs, where physical, chemical, and biological factors impact MSCs’ functions through distinct mechanisms [325]. These mechanisms might involve critical processes like signaling pathway modulation and cellular reprogramming, ultimately influencing MSCs’ capabilities [325]. Illustrating the immunomodulatory role of MSCs exemplifies this phenomenon. During the acute phase or relapse of inflammation, effector T cells secrete pro-inflammatory cytokines, including IFN-γ, TNF, IL-1, and IL-7. These pro-inflammatory cytokines stimulate MSCs to produce substantial amounts of IDO (indoleamine 2,3-dioxygenase) and chemokines. Chemokines serve to attract activated T cells toward MSCs. The elevated concentration of IDO metabolites stemming from this process directly inhibits T cells, resulting in an overall attenuation of the immune response and promotion of tissue repair [1, 326]. On the contrary, in chronic inflammation or during remission, the concentration of anti-inflammatory cytokines, such as TGF-β, increases while pro-inflammatory cytokines decline. Consequently, the production of IDO by MSCs drops below the immunosuppressive threshold. Despite the continued expression of chemokines albeit at lower levels, recruited T cells are not restricted, thus exacerbating the inflammatory immune response [1, 326].

As previously highlighted, MSCs are characterized by their exceptional plasticity. The exclusive focus on purifying MSC subpopulations could potentially impose certain limitations. A more intricate strategy revolves around carefully shaping the extracellular environment of MSCs through deliberate in vitro cultivation, a process terming ‘MSC education’. The objective of this educational initiative is to tap into the inherent variability present within the cell population, steering it towards a consistent manifestation of the intended functions. Across a spectrum of models, diverse categories of educated MSCs have unveiled a range of distinctive functionalities [327–329]. For instance, when BM-MSCs are exposed to WNT5a secreted by gastric cancer cells, a noticeable upregulation of α-SMA expression and an amplified capacity for driving tumorigenesis have been observed [327]. Furthermore, the exosomes released by MSCs primed with neonatal serum have proven capable of expediting the healing of cutaneous wounds by actively stimulating angiogenesis [328]. Notably, MSCs that have undergone a process of education through exposure to chemotherapy have emerged as critical mediators in facilitating communication between MSCs and tumor-initiating cells within specific tumor contexts. This communication is achieved through the selective secretion of cytokines and/or chemokines [329]. In the realm of immune regulation, differently educated MSCs can even exhibit contrasting functionalities. Waterman et al. found that MSCs educated by T-cell signaling (referred to as MSC-I) are primarily geared toward producing pro-inflammatory factors, while MSCs educated by TLR3 signaling (referred to as MSC-II) predominantly express immune-suppressive factors [330]. Similarly, MSCs educated by immune factors such as IFN-γ and TNF-α also demonstrate enhanced immune-suppressive capabilities [1, 331].

To sum up, the development of MSC markers, bolstered by high-throughput techniques, holds substantial potential. Looking at the broader field of MSC research, in addressing the challenge of inconsistent therapeutic efficacy due to MSC heterogeneity, MSC education also presents a viable avenue alongside MSC markers.

## Data Availability

Not applicable.
